# Therapeutic Effect of mRNA SARS-CoV-2 Vaccine on Melanoma Skin Metastases

**DOI:** 10.3390/vaccines10040525

**Published:** 2022-03-28

**Authors:** Dimitrios Bafaloukos, Kalliopi Petraki, Aikaterini Bousmpoukea, Eleni Chatzichristou, Ioannis Pieris, Christos Koutserimpas, George Samonis

**Affiliations:** 1 First Oncology Department, Metropolitan Hospital, Neon Faliron, 18547 Athens, Greece; dimmp@otenet.gr (D.B.); kabousbouke@yahoo.gr (A.B.); hhatzi@gmail.com (E.C.); 2 Department of Pathology, Metropolitan Hospital, Neon Faliron, 18547 Athens, Greece; kalliopipetraki3@gmail.com; 3 Department of Radiology, Metropolitan Hospital, Neon Faliron, 18547 Athens, Greece; ioannispieris@gmail.com; 4 Department of Orthopaedics and Traumatology, “251” Hellenic Air Force General Hospital, 18547 Athens, Greece; chrisku91@hotmail.com

**Keywords:** melanoma, melanoma metastasis, melanoma skin metastasis, antitumor activity, apoptotic activity, mRNA SARS-CoV-2 vaccine

## Abstract

A unique case of multiple metastatic melanoma skin nodules regression in a heavily pretreated, 72-year-old Caucasian female, after administering the second dose of the SARS-CoV-2 mRNA Pfizer-BioNTech vaccine, is presented. Two days after vaccination, all her melanoma skin nodules became painful and were significantly reduced in size. Physical examination and ultrasound imaging confirmed the patient’s observation. The effect was sustained, and further reduction of the nodules occurred after the third vaccine dose. One of the reduced nodules was removed, histologically examined, and its histopathology was compared to that of another such nodule removed and examined earlier. Distinct differences were observed between the two histopathologies, with the most notable the unexpected finding of the absence of infiltrating lymphocytes in the reducer nodule’s melanoma tissue. Based on this observation, the possible immunological mechanism(s) leading to the vaccine’s effect are speculated. More possible is the vaccine’s antitumor and apoptotic activity via stimulation of the Tol Like Receptors 3, 7, and 8, and (downstream) the nuclear factor kappa-light-chain-enhancer of the activated B cells pathway of the non-lymphocytic immune effector cells.

## 1. Introduction

Metastatic malignant melanoma (MMM) is a severe and life-threatening disease, often leading to a fatal outcome. Its frequency is decreasing; however, during the last decade, the disease-free survival and the overall survival of patients suffering MMM have increased due to new effective treatments that include immunotherapy and targeted therapy [1,2].

Immunotherapy involves inhibitory receptors or checkpoint inhibitors. These regimens include cytotoxic T-lymphocyte antigen 4 (CTLA-4) and programmed death-1 (PD-1) protein.

Checkpoint immunotherapy often leads to disease remission of metastatic disease in every visceral organ, including the central nervous system. Combination checkpoint immunotherapy has shown remarkable success, and it is of note that a small number of MMM patients receiving such therapy have been considered cured [1,2]. On the other hand, molecular studies have revealed that melanoma is a tumor with a high mutational burden, and it was understood that mutations could be targeted by drugs. Currently, several agents target the MAPK pathway, while mutations are routinely assessed in these tumors, and, if present, treatment with these agents leads to satisfactory results with long remissions of the disease [1,2,3]. Hence, satisfactory responses of metastatic disease in all visceral organs, including the brain, with the use of BRAF-targeted therapy in cases of melanomas expressing a BRAF V600E mutation, have been observed; while in resistant tumors, targeted therapy with the combination of BRAF and MEK inhibitors resulted in tumor remissions [1,2,3]. BRAF is serine-thionine kinase in the RAS-RAF-MEK-ERK pathway [1,2,3]. 

While immunotherapy and targeted therapy have revolutionized MMM treatment, efforts have been made to develop melanoma vaccines, since vaccination is a type of immunotherapy [4]. Up to now, several vaccines, including those using the mRNA technique, have been used for the treatment of melanoma. These vaccines induce immune responses, although with minimal clinical benefits of limited duration. However, this research is ongoing towards personalized anti-melanoma mRNA vaccines [1,4,5].

The COVID-19 infection that often causes severe acute respiratory syndrome due to coronavirus-2 (SARS-CoV-2) is an ongoing pandemic that has taken millions of lives worldwide and represents a major threat to public health [4,6]. COVID-19 symptoms may differ substantially from patient to patient and may range from undetectable to fatal. The disease represents a special threat for immunocompromised hosts, such as those suffering malignant diseases. Hence, the need for effective treatment, especially for protective vaccines, became urgent [4,7].

The COVID-19 pandemic and the urgent need for a SARS-CoV-2 effective vaccine have led investigators to reassess the value of the mRNA vaccines, and the effort has been proven successful. Hence, the mRNA technology used for vaccines against tumors was ready and shifted rapidly to address the SARS-CoV-2 with success [4,6,7].

The approval of these vaccines was fast, and they have been distributed to many countries since December 2020, aiming to elevate immune response to the COVID-19 virus antigen in humans at risk of developing the infection [6,7]. 

The mRNA SARS-CoV-2 vaccine contains messenger RNA, which causes cells to express the virus spike protein. When the vaccine enters the human body, it helps the immune system identify and destroy the pathogen [4,6,7].

A unique case of metastatic melanoma skin nodules regression, after mRNA Pfizer-BioNTech SARS-CoV-2 vaccination, in a heavily pretreated 72-year-old Caucasian female is presented.

## 2. Case Presentation

We present a patient suffering MMM, with long survival due to several successful treatments.

The patient’s melanoma was firstly diagnosed 10 years ago, when a nevoid malignant melanoma (BRAF mutated), with maxillary lymph node involvement, was removed from her left arm. Consequently, she received adjuvantly interferon for almost a year.

**Figure 1:**
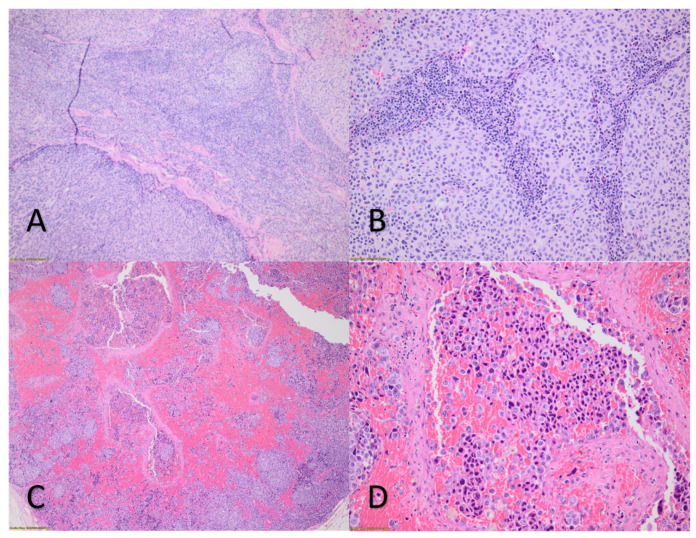
(**A**): Skin nodule—metastatic melanoma before mRNA vaccine. Epithelioid and neoplastic spindle cells. H-E stain at 4×. (**B**): Skin nodule—metastatic melanoma before mRNA vaccine. Lymphocytic infiltration. H-E stain at 20×. (**C**): Skin nodule—metastatic melanoma after mRNA vaccine. Prominent hemorrhagic deposits and necroses. H-E stain at 4×. (**D**): Skin nodule—metastatic melanoma after mRNA vaccine. Marked nuclear atypia and polymorphism—no lymphocytic infiltration. H-E stain at 20× (by conventional light microscope).

However, three years later, the disease recurred with several skin metastatic nodules all over her body. For the following seven years, she has been on several treatments sequentially, receiving practically all existing types of available anti-melanoma therapies (chemotherapy, checkpoint inhibitors, and targeted therapies) [1,2,3]. She started with the dacarbazine and vindesine combination with no response. Then she received ipilimumab with also poor results. Hence, the treatment changed to dabrafenib and, due to intolerance, she was put on pembrolizumab, which kept her disease stable for 18 months. When the disease progressed, she received nivolumab, which also kept her melanoma under control for 15 months (Figure 1A,B).

However, at this point, she developed liver metastasis, and “salvage” treatment was decided using chemotherapy (cis platinum and temozolomide) with poor results. Consequently, because of the BRAF mutated tumor status, she was treated with the encorafenib-binimetinib combination, which led to partial response and stabilization of her melanoma lesions for 18 months. Nevertheless, due to further progression of the skin melanoma nodules, the disease was re-challenged with pembrolizumab with satisfactory results lasting 14 months, up to the present. During these seven years, she never complained of pain.

At this point, 15 days after the last dose of pembrolizumab, she had the first dose of the mRNA-CoV-2 Pfizer-BioNTech vaccine uneventfully. Three weeks later, she received the second vaccine dose. No other medication had been given to her between the two vaccine doses.

Ten hours after the second vaccine dose, all her metastatic skin nodules became progressively painful, and gradually the pain became unbearable, forcing her to seek medical attention. She was hospitalized and treated with analgesics. Two days later, the pain was reduced and finally disappeared. The same day she felt that all her palpable skin melanoma nodules had reduced in size. Physical examination confirmed her observation, since all her skin nodules were significantly smaller by at least 50%. Ultrasound examination of several such nodules confirmed the significant reduction of their size compared to previous recent ultrasound imaging of the same nodules. However, an MRI revealed that the liver metastasis did not show any change.

One week after the skin nodules had been reduced and remained unchanged, one of them was surgically removed and histologically examined. At the same time, the current histopathology was compared to that of a previously removed melanoma nodule (Figure 1C,D).

Notable differences between the previous and the current histopathology were observed. The previous one showed melanoma with high mitotic activity, epithelial and spindle neoplastic cells, and moderate lymphocytic infiltration. The current histopathology confirmed the diagnosis of melanoma, also with high mitotic activity, with neoplastic epithelial cells characterized by pronounced cellular atypia and polymorphism. There were hemorrhagic deposits and areas of necrosis, while lymphocytic infiltration was not observed.

Four months later, when the patient received the third vaccine dose, the metastatic melanoma skin nodules became slightly painful and were reduced a little further in size. However, her visceral disease remained unchanged as previously.

## 3. Discussion

The COVID-19 epidemic became a major risk for patients with neoplastic diseases that are immunocompromised due to their disease and/ or the treatment they receive. Hence, in most developed countries, this patient population has been vaccinated with three doses of the mRNA SARS-CoV-2 vaccine without severe side effects [6,7]. This vaccine is a type of immunotherapy since its administration enhances immunogenicity. This activity is of special interest for patients suffering MMM, for whom immunotherapy represents a major therapeutic tool [4,6,7].

The present patient has been suffering MMM for seven years, and she has received most available treatments, some resulting in partial response and stabilization of her disease [1,2]. For all these years, her performance status was excellent. She had been fully functioning and never complained of pain.

After receiving the second dose of the SARS-CoV-2 mRNA vaccine, her skin metastases became painful and reduced in size within 48 h. The shrinkage of all the melanoma skin nodules had been confirmed by thorough clinical examination and ultrasound imaging.

Considering the existing literature, this is a unique case of special interest, revealing an unexpected activity of this vaccine, indicating that it may exert antitumor activity [5,6,7].

No definite explanation of the vaccine’s antitumor activity can be given. However, some possible mechanisms involved may be speculated upon, based on existing relevant information. It is of interest, however, that initially the mRNA vaccines had been developed as antineoplastic agents [5,6,7].

The lack of lymphocytic infiltration of the reduced metastatic melanoma skin nodule was an unexpected finding. Therefore, it is unlikely that the vaccine triggered an “anamnestic” expansion of preexisting tumor-targeting lymphocytes induced by the previous immunotherapy. High infiltration of the tumor by lymphocytes (TILs) is beneficial in melanoma cases, and the composition of TILs is important [1,2,3]. However, the reduced melanoma nodule of the present patient did not have any TILs. Hence, another possible mechanism may be operative: the exogenous mRNA could have exerted antitumor activity and apoptosis via stimulation of Tol Like Receptor 3 (TLR3), TLR7, and TLR8, and downstream the nuclear factor kappa-light-chain-enhancer of activated B cells (NF-κB) pathway of non-lymphocytic immune effector cells, since TRL7 and TRL8 agonists have been extensively studied as adjunct anti-melanoma drugs [4,8]. The possibility that the observed effects are, perhaps, a delayed response to pembrolizumab treatment cannot be excluded. However, the acute symptoms following the vaccination make this possibility remote. 

The possible salutary effect of the second and third vaccine doses was sustained. Five months after the third vaccination, all skin nodules remained reduced in size, and new skin lesions did not appear.

Irrespectively of the possible immunological mechanism(s) involved, the effect of the SARS-CoV-2 mRNA vaccine on the melanoma metastatic skin nodules, if validated on a larger scale, could have important clinical implications. It is of note that mRNA vaccines directed against patient-specific neoantigens have been used already as melanoma treatment [9,10].

Finally, the lack of response of the liver metastasis is also of interest, as it might reflect differences in tissue-specific response patterns to immune-mediated (presumed catalyzed by the mRNA SARS-CoV-19 vaccine) control of the MMM, as it has been shown in patients with MMM treated with ipilimumab combined with anti-PD-1 therapy [11]. Such individually tailored mRNA cancer vaccine is a topic of active investigation in cancer immunotherapy [12].

Although the mRNA SARS-CoV-2 vaccine has already been given to millions of at-risk patients, new data continue to emerge, and recommendations are updated periodically. There are still immunological results that need to be studied. It must be kept in mind that the urgency to create the vaccine has shortened its development timeline, and though the side-effects are studied extensively, scientific issues regarding the range of the vaccine’s activities are open and require further experience, observation, and analyses of new emerging data.

## 4. Conclusions

This present, signal case, is reported to increase the index of suspicion of oncologists who treat melanoma patients also receiving the SARS-CoV-2 mRNA vaccine, and may present similar effects. Additionally, this report should stimulate further research into the possibility of using the mRNA technology to devise personalized vaccines against each patient’s melanoma, following genetic analysis of each melanoma from a tissue sample of the patient’s tumor.

## Data Availability

Not applicable.
